# Respiratory rate and breathing pattern in dogs and cats in intensive care units and the value of camera surveillance

**DOI:** 10.1111/jsap.13896

**Published:** 2025-06-30

**Authors:** S. di Grado, J. Häggström, A. R. Vestberg, A.‐L. Saraluoma, S. Lundgren, S. Sandström, P. Åhr, B. Liby, J. Strange, L. Wahldén, T. Englund, I. Ljungvall

**Affiliations:** ^1^ Evidensia Södra Djursjukhuset, Kungens Kurva Stockholm Sweden; ^2^ Department of Clinical Science, Faculty of Veterinary Medicine and Animal Science Swedish University of Agricultural Sciences Uppsala Sweden; ^3^ AniCura Bagarmossen Small Animal Hospital Stockholm Sweden; ^4^ Awake Small Animal Hospital Stockholm Sweden; ^5^ Evidensia Uppsala Veterinary Clinic Uppsala Sweden

## Abstract

**Objectives:**

Information on respiratory rate and breathing pattern of dogs and cats in intensive care units is limited. This study aimed to evaluate whether respiratory rate differed in dogs and cats in the intensive care unit when monitored via camera surveillance (Cam+) compared to traditional cage‐side monitoring (Cam−) at various times during the intensive care unit stay, between animals treated for left‐sided congestive heart failure, respiratory diseases and other conditions, and if associations existed between respiratory rate and breathing pattern.

**Materials and Methods:**

Privately owned dogs (*n* = 41) and cats (*n* = 60) treated and monitored in the intensive care units of two small animal hospitals were enrolled in this study. The animals’ respiratory rate and breathing pattern were observed with Cam− and Cam+ throughout their intensive care unit stay.

**Results:**

For both species, median respiratory rate throughout the entire intensive care unit stay was lower when measured with Cam+ (dogs: 30/min, cats: 27/min) compared to Cam− (dogs: 34/min, cats: 31/min) and lower closer to discharge, when observed with both Cam+ (dogs: 22/min, cats: 26/min) and Cam− (dogs: 24/min, cats: 27/min), compared to 1 hour after admission (Cam+; dogs: 38/minutes, cats: 31/minutes and Cam−; dogs: 37/minutes, cats: 36/minutes). Animals with left‐sided congestive heart failure or respiratory disease had higher respiratory rate than those with other conditions and animals with anormal breathing pattern had higher respiratory rate than those with normal breathing pattern.

**Clinical Significance:**

Respiratory rates were influenced by monitoring technique, clinical condition, time point and breathing pattern. Camera surveillance of respiratory rate may be valuable for monitoring treatment outcomes in animals admitted to intensive care unit.

## INTRODUCTION

Monitoring an animal’s respiratory rate (RR) and breathing pattern (BrP) is an essential assessment tool for critically ill dogs and cats. Abnormal RR can occur in animals affected by various conditions, such as primary lung diseases, anaemia‐related disorders and certain cardiac diseases (Domínguez‐Ruiz et al., 2021; Fonfara et al., 2011; Sigrist et al., 2011). Studies on dogs with left‐sided congestive heart failure (CHF) have shown that RR can be a useful clinical parameter for monitoring the effects of treatment (Boswood et al., 2020; Porciello et al., 2016; Schober et al., 2010, 2011). In both healthy dogs and cats and in dogs with cardiac disease, RRs have been shown to be higher at veterinary clinics than when measured in a home environment (Ljungvall et al., 2014; Porciello et al., 2016; Rishniw et al., 2012). Healthy cats were found to have a median RR of 64 breaths/minutes when measured by a veterinarian in a consultation room before the cats were further examined, whereas the median RR was 27 breaths/minutes when measured by the owners in the cats’ home environment (Dijkestra et al., 2018).

To the best of our knowledge, no published studies have systematically investigated RR over time and its association with BrP in dogs and cats in the intensive care unit (ICU). The ICU environment can be stressful for animals, as they are separated from their owners and placed in an unfamiliar setting, which may hinder their ability to sleep or rest. Accordingly, animals in the ICU may have an elevated RR, regardless of disease category or severity, and supporting findings such as BrP may aid in patient assessment. When counting RR, veterinary staff traditionally stand close to the cage to observe chest movements, which may lead to a higher RR than normal for the observed animal. In such situations, monitoring with a closed‐circuit television (CCTV) camera installed in the cage can be helpful. Currently, no published studies compare RR measurements from camera surveillance (Cam+) with those from traditional cage‐side monitoring (Cam−).

This study aimed to evaluate whether RR differed in dogs and cats in the ICU when monitored with Cam+ compared to Cam− over the entire stay, at different times (1 hour after admission and close to discharge) and between animals treated for left‐sided CHF, respiratory disease and other conditions. We also sought to explore potential associations between RR and BrP.

We hypothesised that RR measured with Cam+ would be lower than that with Cam− and that RR will improve over the course of hospitalisation. Furthermore, we hypothesised that RR would be higher in animals with left‐sided CHF or respiratory diseases than in those with other conditions. We also expected higher RR in animals with abnormal BrP than in those with normal BrP.

## MATERIAL AND METHODS

Dogs and cats were recruited for this study from two small animal hospitals in Sweden between May 2021 and June 2022. Owners were informed about the study and signed a consent form allowing their dogs or cats to participate and granting the research group access to their animals’ medical records. The animals included in the study did not undergo any additional procedures, and their treatment and care in the ICU were the same, regardless of study enrolment. As the study was strictly observational, no ethical approval was needed according to the Swedish animal welfare legislation (Swedish Board of Agriculture, n.d.).

### Inclusion and exclusion criteria

Dogs and cats over 1 year old, of any breed and sex, and in need of intensive care were eligible for enrolment. Respiratory rates were measured hourly using both Cam+ and Cam− for at least 4 hours. Animals that were not critically ill (such as those admitted to the ICU for practical reasons, e.g. frequent medications) or spent <4 hours in the ICU were not eligible for the study.

### Camera surveillance

The cameras selected for study met the following practical criteria set by the research group – good image quality, durability, ability to withstand cleaning, adequate performance in various lighting conditions (day and night) and compliance with IT security requirements. Various cameras suitable for this study were reviewed and structurally tested. The Axis P3935‐LR Network Camera was considered adequate for the abovementioned criteria and was chosen for the present study.

**FIG 1:**
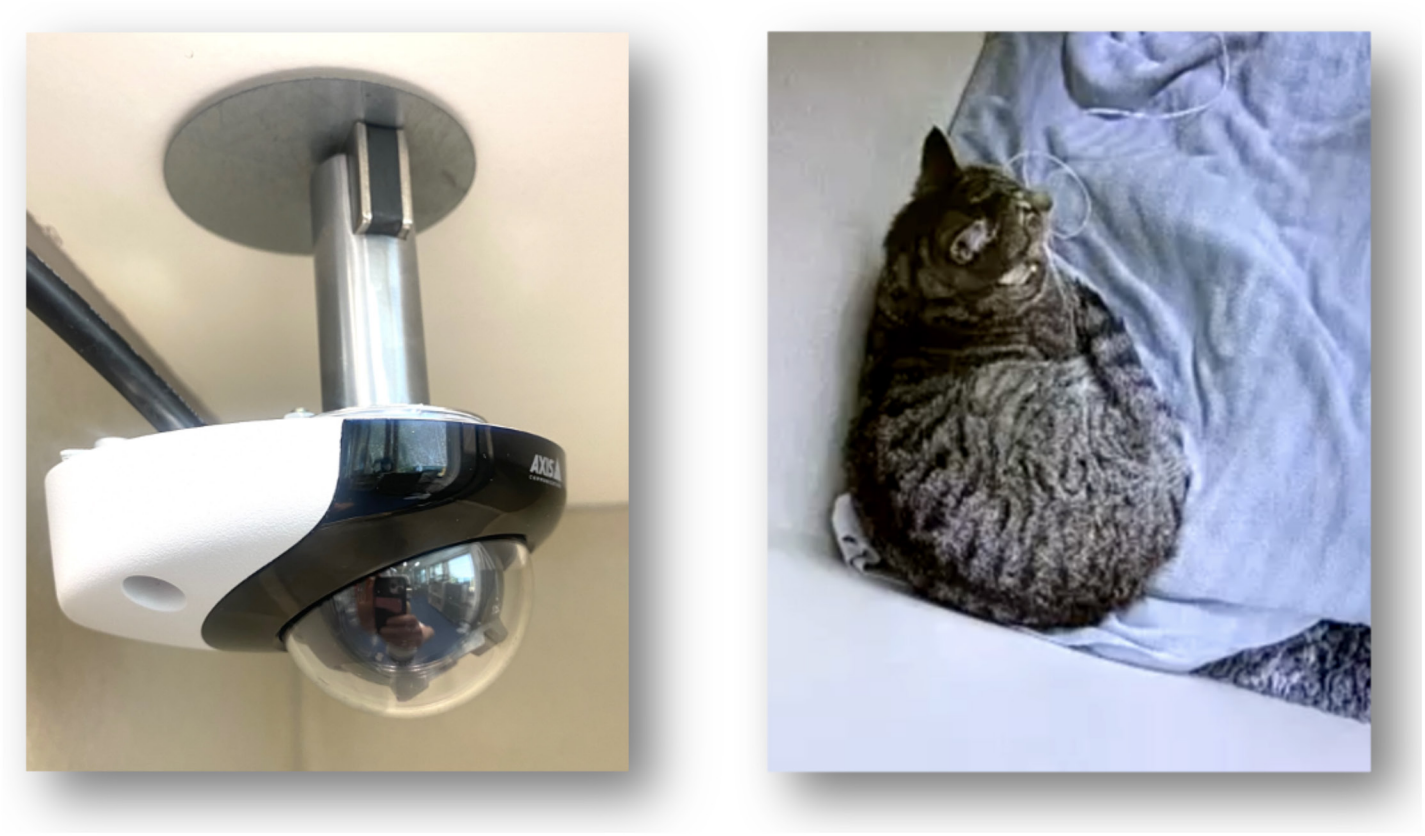
Images illustrating the closed‐circuit television camera unit used in this study, along with an example of camera input for monitoring respiratory rate and breathing pattern on a screen.

Cameras were installed in five cages at one hospital’s ICU, while in the other hospital, a CCTV system was set up in 11 cages, allowing the cameras to be moved between different cages (see Fig 1). The cameras were connected to the hospitals’ Wi‐Fi systems, and live video recordings were viewed on a computer screen in the ICU. All video recordings of the dogs and cats enrolled in the study were stored on an external hard drive.

### Study protocol

A protocol was designed in which details including species (dog or cat), breed, age, sex, body weight (BW), general condition, case history, presenting signs, RR, BrP and final clinical diagnosis were noted by the attending staff in the ICU for each enrolled animal. The medical record of each animal was reviewed by research team members (SDG and IL) at a later time to verify the data recorded in the protocol. The team also categorised each animal’s clinical diagnosis based on the organ system responsible for the clinical signs leading to ICU admission, assigning the animals to one of the following groups: (1) left‐sided CHF, (2) respiratory diseases or (3) other conditions.

To facilitate the recruitment process and inform staff, flowchart diagrams and written documents about the study protocol were posted on the walls of the ICU and emergency departments at both hospitals.

### RR and BrP assessments

One hour after ICU admission, while the animal was in its cage, the attending staff visually assessed the RR for 1 minute via a CCTV device connected to computer screens in the ICU office (Cam+). Within the next min, the same staff assessed RR and BrP visually from a short distance away from the animal’s cage (Cam−). The staff were encouraged to minimise stress on the animal by avoiding standing too close to the cage and maintaining silence during the evaluation. The BrP was subjectively classified as normal or abnormal at the discretion of an observer (staff member). Abnormal BrP was defined as increased effort during one or both phases of breathing, a marked difference in duration between the two phases, and/or paradoxical thoracic and abdominal movements during respiration (Domínguez‐Ruiz et al., 2021). Before the study began, staff involved in the study were shown videos of dogs and cats with abnormal BrP as examples.

All staff involved in assessing RR and BrP were instructed to monitor the animal while it was sleeping or resting, if possible. If the animal had recently been out of its cage, or if the animal was panting, staff were advised to wait until the animal seemed to settle in its cage before evaluating RR and BrP. For all animals, both RR and BrP assessments were repeated hourly for the first 4 hours after ICU admission and thereafter every third hour until the animal was close (within 1 hour) to being discharged, euthanased or had died. The final observation, regardless of event, is hereafter referred to as ‘close to discharge’. All results were recorded in the animal study protocol for the entire ICU enrolment period.

For quality assessment, all video recordings were evaluated by a veterinarian (SDG) experienced in cardiology and critical care to verify the RR and BrP classifications previously made by the staff. If there was a discrepancy between the staff’s classification and the video recordings, the video‐based classification was used.

### Statistical analysis

Statistical analyses were performed using JMP Pro® 16.0.0. Descriptive statistics were used to analyse the age, BW, disease group, RR and BrP. Group data for dogs and cats are presented as median values and interquartile ranges (IQR). Normal distribution of included variables was evaluated by inspection of QQ plots and differences in variance between groups were tested using the *F*‐test. Statistical significance was set at P < 0.05, unless otherwise specified.

The Wilcoxon signed‐rank test was performed to examine changes in RR that each animal experienced when monitored via Cam+ or Cam− from 1 hour after admission to close to discharge, both for the entire group of animals and for dogs and cats separately. Two curves were constructed for each dog and cat – one for RR measured with Cam+ and one for the RR measured with Cam−. RRs were plotted on the *y*‐axis, with time after inclusion in the study on the *x*‐axis. The area under the curve was calculated for each curve, and the sum was divided by the total time (hour) that the dog or cat was observed from the first to the last observation (Matthews et al., 1990). This calculation yielded a sum value of the measured RR, representing the average for each animal over the entire observation period. All observations were included, and the result was independent of the duration of the animal’s observation (Boswood et al., 2018; Häggström et al., 2013). These sum values were then used in the statistical analyses.

The Kruskal–Wallis test was used to examine whether RR values differed when monitored with Cam+ or Cam− 1 hour after admission and close to discharge, for all animals, as well as for dogs and cats separately. The same test was also used to examine potential differences in RR values between the three disease groups 1 hour after admission and close to discharge. If a significant difference (P < 0.05) was found, the Wilcoxon rank‐sum test was performed. The Bonferroni correction was used for group comparisons when the number of groups included in the analysis was more than two.

The Wilcoxon rank‐sum test was used to examine possible differences in RR between animals with abnormal BrP and those with normal BrP, 1 hour after admission, for dogs and cats separately. Data were assessed by combining information from both Cam+ and Cam− for RR and BrP.

## RESULTS

### Animals

A total of 101 animals were included in the study – 41 dogs and 60 cats. The dogs had a median age of 5.7 years (IQR 2.5 to 11 years) and a median BW of 10 kg (IQR 5.0 to 19.5 kg). Dogs of mixed breeds (*n* = 7) were the most commonly recruited, followed by four miniature schnauzers, three Chihuahuas and three Rottweilers. In addition to these breeds, another 20 breeds with one to two dogs in each group were included in this study. The cats had a median age of 6.3 years (IQR 2.1 to 10.7 years) and a BW of 4.6 kg (IQR 3.6 to 5.4 kg). Domestic short‐/long‐haired (*n* = 33) cats were the most commonly recruited cats, followed by six Maine coons, three Bengals and three Norwegian forest cats. In addition, another ten breeds with one to two cats in each group were included in this study. A total of 26 dogs and 33 cats were included in the other condition group, eight dogs and 14 cats in the respiratory disease group and seven dogs and 13 cats in the left‐sided CHF group (see Table 1).

**Table 1:** Number of included dogs and cats grouped by disease category

Disease category	Dogs (41)	Cats (60)
Left‐sided congestive heart failure	**7**	**13**
Respiratory diseases	**8**	**14**
Other conditions	**26**	**33**
Gastrointestinal disease	6	6
Neurological disease	8	8
Urinary tract disease	1	9
Hematologic/immunologic	3	4
Intoxication	4	3
Neoplastic disease	2	1
Musculoskeletal disease	1	1
Reproductive disease	1	1

n Number

The bold values represent the main disease categories, whereas the numbers that are not in bold are the sub‐categories in the “other condition” group.

Dogs enrolled in the study were hospitalised for a median time of 24.00 hours (IQR 12.50 to 41.90 hours) and cats for a median time of 25.60 hours (IQR 12.90 to 50.50 hours). Of the enrolled animals, 6 of 35 (17%) dogs and 12 of 48 (25%) cats were euthanased or had died during ICU admission.

### Respiratory rates

**FIG 2:**
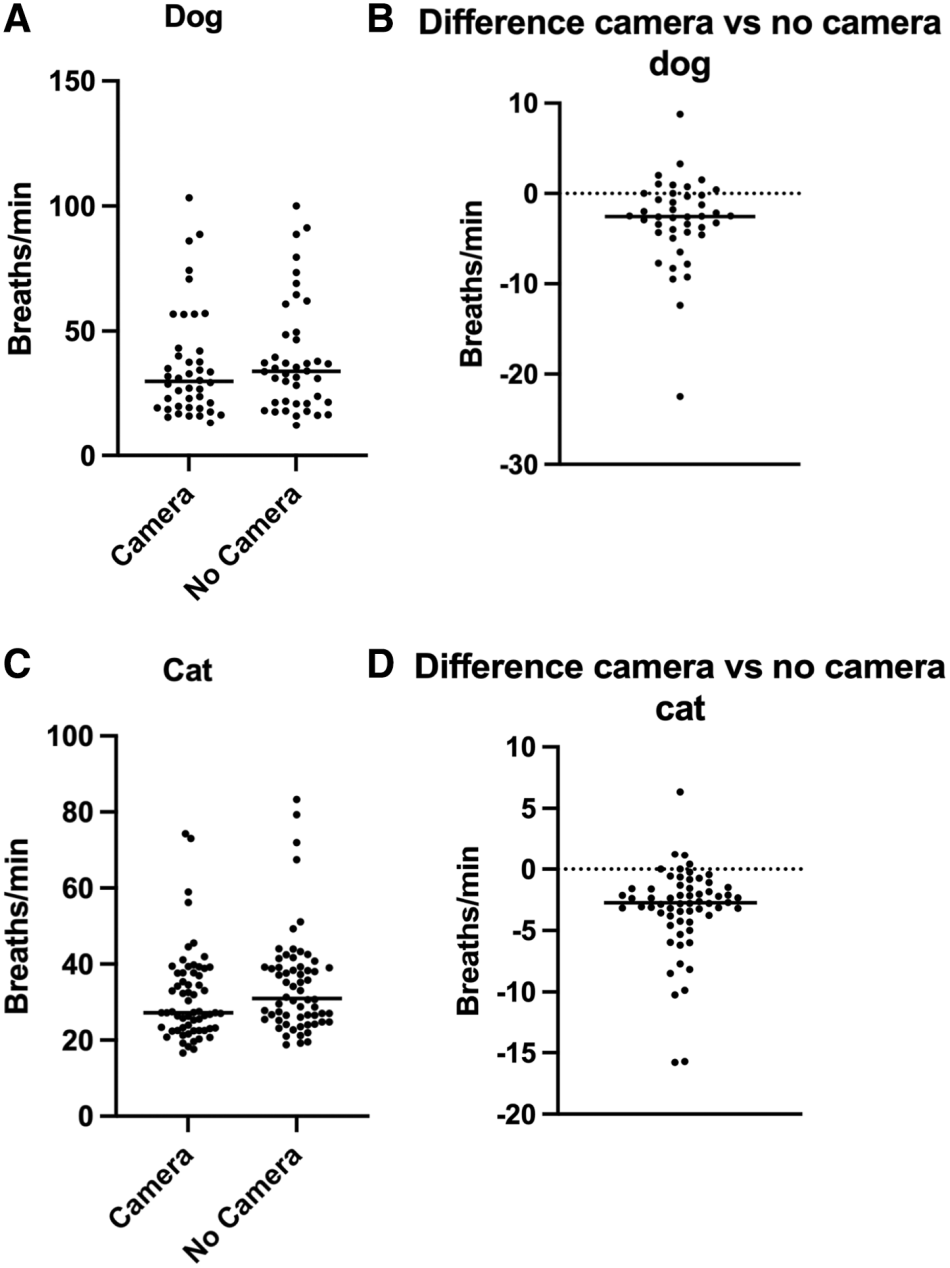
Scatterplots showing the distribution of respiratory rate (RR) for dogs (*n* = 41) and cats (*n* = 60), respectively, throughout the entire intensive care unit (ICU) stay, measured via camera monitoring (Cam+) and traditional cage‐side monitoring (Cam−) (A, C) and the difference between Cam+ and Cam− for each dog (B) and cat (D). RR for each dog was calculated as the area under the curve (AUC)/number of hours at ICU. Bars indicate the median value. The median RR was lower during the ICU stay when measured with Cam+ (dogs: 30/minutes (IQR 19 to 42), cats: 27/minutes (IQR 23 to 37) compared to Cam− (dogs: 34/minutes (IQR 21 to 49), cats: 31/minutes (IQR 25 to 39); P‐value: all <0.0001.

The median RR during the entire ICU stay was lower for both dogs and cats, respectively, when measured using Cam+ compared to Cam−, with the median difference for dogs being 2.5 breaths/minutes (IQR 0.08 to 4.3) and for cats 2.9 breaths/minutes (IQR 1.5 to 3.9), all P < 0.0001 (see Fig 2).

**FIG 3:**
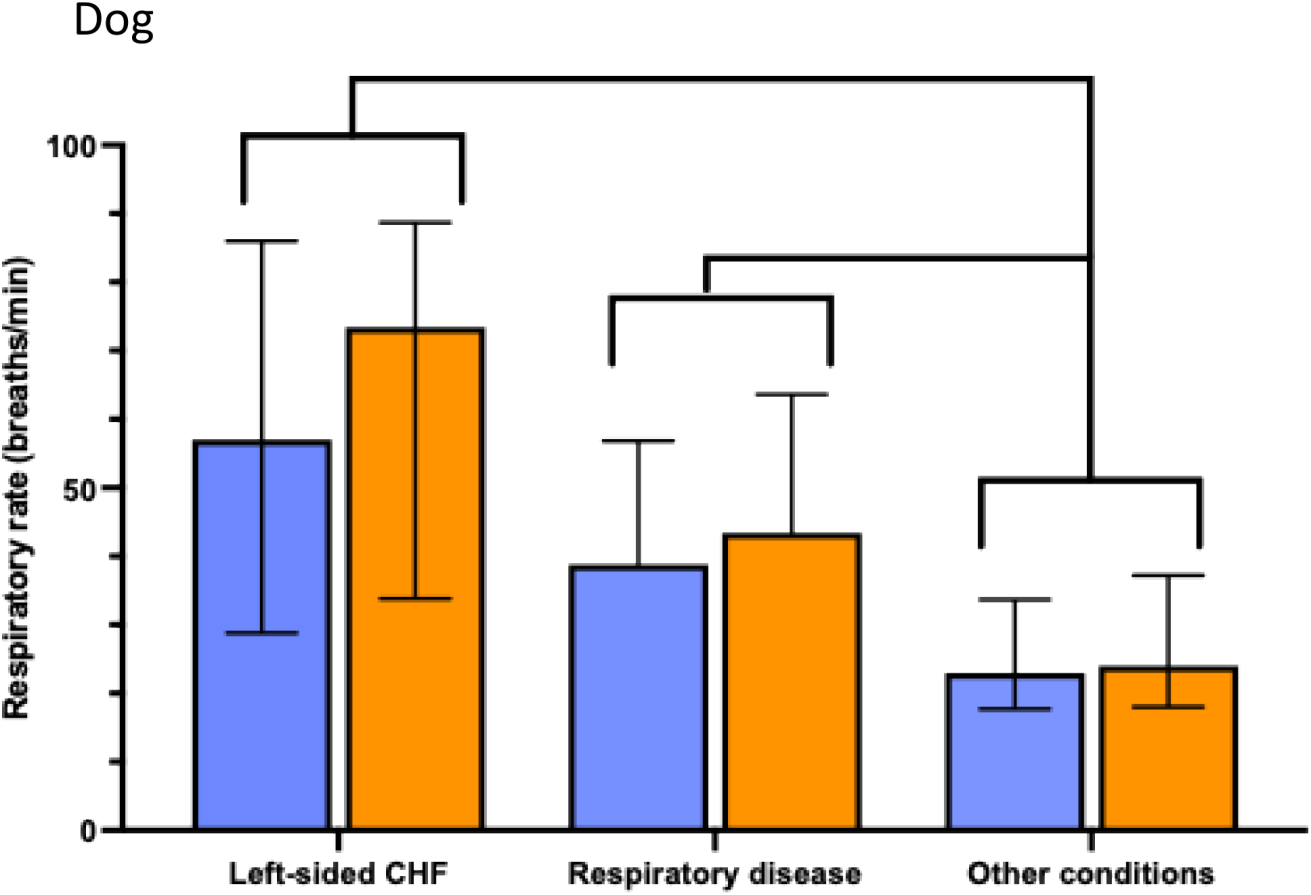
A bar chart showing median respiratory rate (RR) and interquartile range (IQR) throughout the entire intensive care unit (ICU) stay for dogs, grouped by diagnosis. Blue bars indicate measurement with camera monitoring (Cam+), and orange bars represent traditional cage‐side monitoring (Cam−). RR for each dog was calculated as the area under the curve (AUC)/number of hours at ICU. A P‐value of < 0.017 was considered significant after Bonferroni correction. RR was higher in dogs with left‐sided congestive heart failure (Cam+, P = 0.003; Cam− P = 0.002) or respiratory diseases (Cam+, P = 0.004; Cam−, P = 0.005) compared to that in dogs with other conditions.

**FIG 4:**
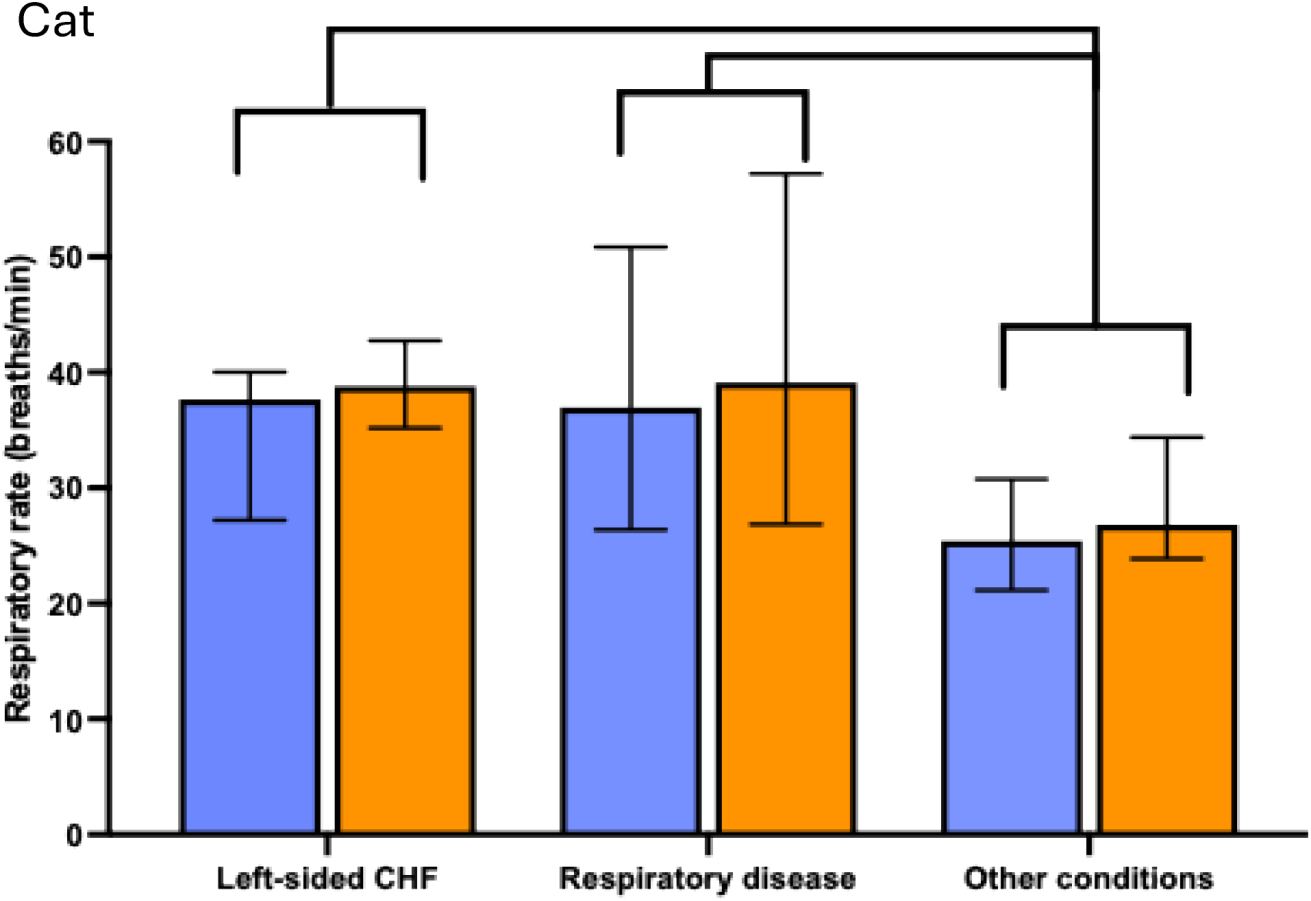
A bar chart illustrating median respiratory rate (RR) and interquartile range (IQR) throughout the entire intensive care unit (ICU) stay for cats, grouped by diagnosis. Blue bars indicate measurement with camera monitoring (Cam+), and orange bars represent traditional cage‐side monitoring (Cam−). RR for each cat was calculated as the area under the curve (AUC)/number of hours at ICU). A P‐value of < 0.017 was considered significant after Bonferroni correction. RR was higher in cats with left‐sided congestive heart failure (Cam+ P = 0.0008, Cam− P = 0.0006) or respiratory diseases (Cam+ P = 0.002, Cam− P = 0.006) compared to that in cats with other conditions.

The median RR during the entire ICU stay was higher in dogs diagnosed with left‐sided CHF (Cam+, P = 0.003; Cam−, P = 0.002) or respiratory disease (Cam+, P = 0.004; Cam−, P = 0.005) than in those diagnosed with other conditions (see Fig 3). The median RR during the entire ICU stay was higher in cats diagnosed with left‐sided CHF (Cam+; P = 0.0008, Cam−; P = 0.0006) or respiratory diseases (Cam+; P = 0.002, Cam−; P = 0.006) than in those diagnosed with other conditions (see Fig 4).

The RRs measured 1 hour after admission to the ICU and close to discharge, monitored using Cam+ and Cam−, are summarised in Table 2 for all animals grouped together, as well as for dogs and cats separately, and for animals diagnosed with left‐sided CHF, respiratory diseases or other conditions. The RR was lower close to discharge compared to 1 hour after admission for the entire group of animals, as well as for dogs and cats separately, regardless of whether the RR was measured with Cam+ or Cam− (all P < 0.001). The RR was higher for dogs with left‐sided CHF when assessed 1 hour after admission (Cam+; P = 0.014) and close to discharge (Cam+; P = 0.003, Cam−; P = 0.006) compared to that in those diagnosed with other conditions. No statistical differences in RR when monitored by Cam+ or Cam− were observed between the other disease groups at these assessment occasions. Furthermore, the RR was higher 1 hour after admission for cats with left‐sided CHF (Cam+, P = 0.0004; Cam, P = 0.003) or respiratory diseases (Cam+, P = 0.003; Cam−, P = 0.004) than in those diagnosed with other conditions. No statistical differences in RR when monitored via Cam+ or Cam− were observed among the three disease category groups when assessed close to discharge.

**Table 2:** Median and interquartile range (IQR) of RR (breaths/min) for the included animals, measured with Cam+ and Cam− 1 hour after ICU admission and close to discharge

	All animals (*n* = 101)		Dogs (*n* = 41)		Cats (*n* = 60)	
	Cam+	Cam−	Cam+	Cam−	Cam+	Cam−
All diagnoses						
1 hour after admission	34 (24 to 56)^1a^	36 (27 to 60)^1b^	38 (28 to 66)^1a^	37 (27 to 68)^1a^	31 (24 to 56)^1a^	36 (26 to 56)^1b^
Close to discharge	24 (20 to 32)^2a^	26 (16 to 36)^2b^	22 (16 to 33)^2a^	24 (16 to 35)^2b^	26 (22 to 32)^2a^	27 (24 to 36)^2b^
Left‐sided CHF						
1 hour after admission	43 (34 to 77)^1a^	46 (35 to 70)^1a^	77 (32 to 110)^1a^	69 (32 to 114)^1a^	40 (34 to 60)^1a^	42 (36 to 66)^1a^
Close to discharge	29 (26 to 36)^2a^	32 (27 to 46)^2a^	44 (26 to 76)^1a^	64 (34 to 77)^1a^	28 (24 to 32)^2a^	28 (26 to 34)^2a^
Respiratory disease						
1 hour after admission	52 (28 to 68)^1a^	56 (28 to 74)^1a^	50 (39 to 64)^1a^	63 (42 to 111)^1a^	54 (28 to 71)^1a^	56 (28 to 73)^1a^
Close to discharge	26 (21 to 40)^2a^	28 (21 to 44)^2a^	20 (19 to 52)^1a^	21 (20 to 60)^2a^	28 (24 to 40)^2a^	30 (24 to 43)^2a^
Other conditions						
1 hour after admission	28 (20 to 38)^1a^	30 (22 to 40)^1b^	30 (20 to 41)^1a^	31 (22 to 41)^1a^	28 (20 to 35)^1a^	29 (22 to 40)^1a^
Close to discharge	23 (19 to 30)^2a^	25 (20 to 34)^2b^	20 (14 to 24)^2a^	20 (14 to 28)^2a^	24 (20 to 31)^1a^	27 (24 to 37)^1b^

The results are presented for all animals grouped together and for dogs and cats separately. Comparisons were made between measurements with Cam+ and Cam− at 1 hour after admission and close to discharge for all diagnoses grouped together and for left‐sided CHF, respiratory disease and other conditions separately. Letters indicate comparisons in a row and numbers comparisons in columns. A P‐value <0.05 was considered significant. Measurements sharing the same superscript letter or number were not significantly different

Cam− traditional cage‐side monitoring, Cam+ Video camera surveillance, CHF congestive heart failure, n Number, RR Respiratory rate

### Breathing pattern

RR measured 1 hour after ICU admission was higher in dogs with abnormal BrP (*n* = 21) than in those with normal BrP (*n* = 20) when measured with Cam+ (P = 0.034), but not with Cam−. The RR measured 1 hour after ICU admission was also higher in cats (*n* = 30) with abnormal BrP than in those with normal BrP (*n* = 30, Cam+, P = 0.0072; Cam−, P = 0.0053). All animals showed normal BrP before ICU discharge.

## DISCUSSION

This study demonstrates that RR in dogs and cats hospitalised in the ICU is influenced by monitoring technique, clinical condition, time point and BrP: Cam+ recorded lower median RR compared to Cam−, animals with left‐sided CHF or respiratory disease had higher RR than those with other conditions, regardless of monitoring technique, RR decreased from admission to discharge and was lower in animals with a normal BrP.

The RR was consistently lower for both dogs and cats when measured with Cam+ compared to Cam−; during the entire stay, 1 hour after admission and at discharge from ICU. A likely explanation is that the type of monitoring technique, Cam+ *versus* Cam−, may induce different levels of situational stress for the animal, even though staff were encouraged to optimise all assessment situations. During Cam− assessments, staff had to stand closer to the animal’s cage, which could have contributed to higher stress and, consequently, a higher RR compared to when the assessment was performed using Cam+. Although statistically significant, the differences between Cam+ and Cam− RR values were generally small. However, as with all clinical studies, centricity measurements do not represent every individual. For each patient, the monitoring technique may have a potential impact on the assessment outcome.

Dogs and cats diagnosed with left‐sided CHF or respiratory disease had an overall higher RR than those with other conditions when measured using Cam+ or Cam−. These findings can be explained by the pathophysiological mechanisms associated with diseases affecting the respiratory system. Left‐sided CHF leads to fluid accumulation in the alveoli, resulting in increased RR and laboured breathing due to hypoxia (Gehlbach & Geppert, 2004). Respiratory diseases, such as pleural space disease, also lead to higher RR as the lungs cannot expand normally, making respiration less efficient. Despite the likely influence of stress on this clinical parameter in the ICU environment, the results from this study suggest that both disease status – including disease category and severity (whether the animal is stabilised or not) – influence the RR values obtained when monitoring animals in such settings.

The RR was higher 1 hour after admission compared to the last observation close to discharge for all groups of dogs and cats enrolled in the study, as well as for animals diagnosed with left‐sided CHF or respiratory disease, regardless of whether RR was measured with Cam+ or Cam− (Table 2). Overall, the differences in the RR values between monitoring occasions were more pronounced in cats than in dogs. This information may help clinicians assess treatment response and determine the optimal time for discharge. There was no difference in RR values obtained with Cam+ or Cam− close to discharge for dogs or cats with respiratory or cardiac disease, suggesting that the animals may have become accustomed to being monitored by the staff.

The median RR before discharge from the ICU when measured using Cam− was 24 breaths/minutes (IQR 16 to 24 breaths/min) for dogs and 28 breaths/minutes (IQR 24 to 37 breaths/min) for cats. These findings may be of value, as no previous studies have assessed the RR in a standardised manner for dogs and cats in the ICU prior to discharge. Notably, the median RR before discharge in our study sample was derived from a diverse group of animals with varying diagnoses and disease severities and not all animals were sleeping or resting at the time of assessment. The mean RR values for dogs and cats with medically controlled left‐sided CHF, when observed in their home environments, have been reported to be 24 breaths/minutes for both dogs (IQR 12 to 44 breaths/min) and cats (IQR 15 to 45 breaths/min) (Porciello et al., 2016), which is comparable to the results at discharge for the dogs and cats investigated in our study. No published studies have to date, to our knowledge, investigated RR in dogs and cats with respiratory disease, either in clinical settings or in the home environment. The results of the present study indicate that RR can be used as a clinical parameter to monitor disease progression and response to therapy in dogs and cats admitted to the ICU, as it decreased markedly for many animals once stabilised, especially in those with diseases affecting the respiratory organ. As some dogs and cats had RR exceeding 30 breaths/minutes before discharge (measured with Cam+ and Cam−), additional clinical variables, such as BrP, must also be considered when deciding whether an animal is ready for discharge from the ICU. The RR may increase due to stress or pain, and BrP may help refine clinical assessment. Indeed, RR was higher at admission in dogs (Cam+) and cats (both Cam+ and Cam−) with abnormal BrP compared to those with normal BrP.

A limitation of this study was the diversity of the study population, comprising dogs and cats in need of intensive care, with varying diagnoses and disease severities. Furthermore, animals in this study were treated with a variety of drugs at different doses and underwent various procedures and handling at different occasions, none of which were controlled for in the statistical analyses. However, as this was a clinical study intended to mimic animals admitted to ICU, the authors believe the results for RR are still representative for dogs and cats with various diseases admitted to ICU.

Additionally, this study was conducted in clinical settings, where variables such as sounds made by other animals or staff in the ICU could not be fully controlled and may have negatively influenced the level of stress for the animals being evaluated. A further limitation was that the fear anxiety stress score was not included in the study protocol. The experience of the staff working in the ICU may have also varied, which could have influenced the RR and BrP results. For quality assessment, all video recordings were evaluated at a later occasion by a veterinarian (SDG) experienced in cardiology and critical care to verify assessments previously made by the staff. The present study was exploratory in nature and because of the lack of previously published data, statistical power calculations could not be performed to determine the sample size.

In conclusion, the median RR during the entire ICU stay was lower for cats and dogs when measured with Cam+ than with Cam−. Moreover, RR was lower closer to discharge than 1 hour after ICU admission. The RRs were higher in dogs and cats with left‐sided CHF or respiratory diseases than in those with other conditions. Furthermore, dogs and cats with abnormal BrP had a higher RR than those with normal BrP. These findings highlight the importance of context and monitoring technique when assessing RR in critically ill dogs and cats, and the result may help optimise assessment of RR in critically ill animals in the ICU.

### Author contributions

**S. di Grado:** Conceptualization (lead); data curation (lead); formal analysis (lead); funding acquisition (lead); investigation (lead); methodology (lead); project administration (lead); resources (supporting); software (lead); visualization (lead); writing – original draft (lead). **J. Häggström:** Formal analysis (equal); methodology (supporting); supervision (supporting); visualization (equal); writing – review and editing (supporting). **A. R. Vestberg:** Funding acquisition (equal); methodology (supporting); project administration (equal); writing – review and editing (supporting). **A.‐L. Saraluoma:** Investigation (supporting); methodology (supporting); project administration (supporting); software (supporting); writing – review and editing (supporting). **S. Lundgren:** Investigation (supporting); methodology (supporting); software (supporting); writing – review and editing (supporting). **S. Sandström:** Investigation (supporting); software (supporting); writing – review and editing (supporting). **P. Åhr:** Investigation (supporting); project administration (supporting); software (supporting); writing – review and editing (supporting). **B. Liby:** Investigation (supporting); project administration (supporting); writing – review and editing (supporting). **J. Strange:** Investigation (supporting); software (supporting); writing – review and editing (supporting). **L. Wahldén:** Investigation (supporting); software (supporting); writing – review and editing (supporting). **T. Englund:** Investigation (supporting); software (supporting); writing – review and editing (supporting). **I. Ljungvall:** Conceptualization (lead); data curation (equal); formal analysis (lead); funding acquisition (lead); investigation (lead); methodology (lead); project administration (lead); resources (equal); software (lead); supervision (lead); visualization (equal); writing – original draft (equal); writing – review and editing (lead).

### Conflict of interest

No conflicts of interest have been declared.

## Data Availability

The data from this study are available in anonymised format only from the corresponding author upon reasonable request.
